# Interruption of the tricarboxylic acid cycle in *Staphylococcus aureus* leads to increased tolerance to innate immunity

**DOI:** 10.3934/microbiol.2021031

**Published:** 2021-12-15

**Authors:** Alexis M. Hobbs, Kennedy E. Kluthe, Kimberly A. Carlson, Austin S. Nuxoll

**Affiliations:** Department of Biology, University of Nebraska at Kearney, 2401 11^th^ Ave, Kearney, NE 68849, USA

**Keywords:** *Staphylococcus aureus*, *Drosophila melanogaster*, antimicrobial peptides, persisters, tolerance

## Abstract

*Staphylococcus aureus* is widely known for its resistance and virulence causing public health concerns. However, antibiotic tolerance is also a contributor to chronic and relapsing infections. Previously, it has been demonstrated that persister formation is dependent on reduced tricarboxylic acid (TCA) cycle activity. Persisters have been extensively examined in terms of antibiotic tolerance but tolerance to antimicrobial peptides (AMPs) remains largely unexplored. AMPs are a key component of both the human and *Drosophila* innate immune response. TCA cycle mutants were tested to determine both antibiotic and AMP tolerance. Challenging with multiple classes of antibiotics led to increased persister formation (100- to 1,000-fold). Similarly, TCA mutants exhibited AMP tolerance with a 100- to 1,000-fold increase in persister formation when challenged with LL-37 or human β-defensin 3 (hβD3). The ability of TCA cycle mutants to tolerate the innate immune system was further examined with a *D. melanogaster* model. Both males and females infected with TCA cycle mutants exhibited increased mortality and had higher bacterial burdens (1.5 log) during the course of the infection. These results suggest increasing the percentage of persister cells leads to increased tolerance to components of the innate immune system.

## 1. Introduction

*Staphylococcus aureus* is responsible for a multitude of infections, varying from minor skin and soft tissue infections to more complicated illnesses, such as bacteremia or endocarditis [1]. Recurring *S. aureus* bacteremia occurs in 10% of patients and can be as high as 15%, despite the use of antibiotics [2]–[4]. Current antibiotics are largely ineffective against chronic bacterial infections. Fifty percent of all infections are caused by biofilms [5]. These infections are extremely difficult to eradicate, and surprisingly, are often caused by drug-susceptible pathogens. It has been suggested this phenomenon is largely due to persister cells surviving antibiotic challenge within a biofilm [6],[7]. Persisters are traditionally defined as a phenotypic variant exhibiting a high degree of metabolic inactivity and tolerance to antibiotics [6],[8]. Tolerance, unlike resistance, is not passed on to progeny and is growth phase dependent. Recent work has elucidated the underlying mechanism of persister formation in *S. aureus*, but it remains unknown as to whether persisters have an advantage in evading the host immune response.

Most of our knowledge on the interaction between persisters and the immune system comes from studies performed in *Salmonella*. In these studies, *Salmonella* persisters are able to skew macrophage polarization from a classically activated (M1) to an alternatively activated (M2) response promoting persister survival within the intracellular milieu [9]. Additionally, *S. aureus* biofilms also skew the macrophage response towards those with the M2 polarization, thus promoting persistence rather than clearance [10]. These studies support the idea of the immune system interacting with *S. aureus* persisters differently than non-persister counterparts, however direct evidence is lacking. Recent work in *S. aureus* has demonstrated that reactive oxygen species (ROS), which are often generated from cells closely associated with innate immunity, induce persister formation [11]. Additionally, *S. aureus* cells surviving antibiotic challenge within a macrophage were found to be persister cells [12]. Despite recent work demonstrating persisters are induced upon ROS exposure and survive within the macrophage during antibiotic challenge, it is unclear whether persisters have increased survival to innate immune components.

Persister formation in *S. aureus* has been associated with decreased tricarboxylic acid (TCA) cycle activity, which consequently depletes intracellular ATP [8],[13]. Decreases in ATP likely affect the antibiotic targets within the bacterial cell, decreasing the cidal action exhibited by many antibiotics. Antimicrobial peptides (AMPs) are essential to the innate immune response and often share similar targets as antibiotics. Human AMPs that help control *S. aureus* dissemination include LL-37 and human β-defensin 3 (hβD3). LL-37 is found in the lysosome of macrophages and polymorphonuclear leukocytes (PMNs) and shares a similar antimicrobial activity with the antibiotic daptomycin [14]. LL-37 works by inserting into and forming pores in both the inner and outer membrane of the bacteria, thus disrupting their integrity [15]. The AMP, hβD3, is produced by epithelial cells and neutrophils and shares a similar antimicrobial activity with the antibiotic vancomycin, which works by targeting membrane-bound precursor lipid II [16]. The similarities shared by antibiotics and AMPs makes it plausible that persisters would also show tolerance to AMPs in addition to antibiotics. AMPs are commonly one of the first defenses to an invading pathogen and produced by most multicellular organisms, including invertebrates.

*Drosophila melanogaster*, the common fruit fly, is considered a valuable tool for studying aspects of the vertebrate innate immune system. This is largely due to homology in genes associated with the innate immune system, in which ~80% of human immunity genes have homology within *D. melanogaster* [17]. Unlike vertebrates that have innate and adaptive immune response, *D. melanogaster* only contain an innate immune system [18]. This lack of an adaptive immune component allows for teasing apart whether persisters tolerate the innate immune response better than non-persister cells. Innate immunity is relatively conserved between vertebrates and invertebrates, including AMPs with *D. melanogaster* having both defensins and cathelicidins.

The objectives of this study were to determine if TCA cycle mutants exhibit increased persister formation. From there, the interactions between the TCA cycle mutants and the innate immune system were further investigated, both when treated with AMPs *in vitro* and in a *Drosophila* sepsis infection.

## 2. Materials and methods

### 2.1. Bacterial strains and growth conditions

*S. aureus* strains used in this study were all derived from HG003, and all bacteria were grown in Tryptic Soy Broth (TSB) at 37 °C at 225 rpm. Defined *bursa aurealis* transposon mutants were acquired from the Nebraska transposon mutant library (transposon insertions designated by ::*NΣ*, Biodefense and Emerging Infections [BEI] Research Resources Repository, Manassas, VA). Transposon mutants were transduced into HG003 from LACJE2 using bacteriophage φ11.

### 2.2. Time-dependent kill (persister) assays

Overnight cultures were grown to mid-exponential phase in 12.5 mL TSB (125 mL) flask and challenged with either ciprofloxacin, oxacillin, or daptomycin (10, 10, 200 µg/mL, respectively). In the daptomycin assay, 50 µg/mL CaCl_2_ was added to ensure antibiotic activity. At each time point, 100 µL of culture was removed, pelleted by centrifugation, washed with 1% NaCl, serial diluted, and plated onto Tryptic Soy Agar (TSA) to enumerate surviving cells. As a control for survival defects, cultures were maintained in TSB and surviving cells were enumerated alongside treated cultures. For antimicrobial peptide time-dependent kill assays, cultures were grown to mid-exponential phase, centrifuged, resuspended in 1X phosphate buffered saline (PBS), and diluted again 1:25 in 1X PBS. Cultures were maintained in PBS due to the salt sensitivity of the antimicrobial peptides [19]. Following resuspension and dilution, cultures were challenged with either LL-37 or hβD3 (2.5 µg/mL and 5 µg/mL, respectively). Cultures were also maintained in 8 µg/mL chloramphenicol to prevent regrowth of resistant mutants. As a control for growth, cultures were also maintained only in the presence of chloramphenicol and survivors were enumerated alongside treated cultures.

### 2.3. Dissipation of membrane potential

To confirm membrane potential is needed for daptomycin and LL-37 antimicrobial activity, the BacLight Bacterial Membrane Potential Kit (Invitrogen™ B34950, Waltham, MA) was used according to manufacturer's instructions in combination with flow cytometry. Cultures were grown to exponential phase and DiOC_2_ was excited at 488 nm. Green and red fluorescence was detected with bandpass filters of 515/20- and 616/23-nm, respectively. As a positive control, carbonyl cyanide *m*-chlorophenylhydrazone (CCCP), a proton-ionophore, was added to dissipate membrane potential.

Once CCCP was confirmed to dissipate membrane potential, persister assays were performed as above with the following modifications. Following growth to mid-exponential phase (~4.5 h), carbonyl cyanide m-chlorophenylhydrazone (CCCP), a proton-ionophore, was added to dissipate membrane potential. CCCP was added at 20 µM for 10 minutes prior to antibiotic or antimicrobial peptide challenge [20]. After 10 minutes, cultures were centrifuged and washed twice with fresh media. Bacterial survival was enumerated over 72 h and 18 h for cells treated with daptomycin and LL-37, respectively.

### 2.4. Drosophila melanogaster husbandry and septic infection

All adult files used were 1–2 days old and were reared on standard banana food at 25 °C with a diurnal light cycle. Flies were watched closely once pupation was evident. At the time of eclosion, flies were lightly etherized, separated by sex, and allowed to age for 24 hours. After maturing for 24 hours, flies were infected by septic pinprick as previously described [21]. Briefly, flies were anaesthetized with ether and pierced in the thorax with a Tungsten dissecting needle with a diameter of 1µm (Roboz Surgical Instrument Co, RS-6063, Gaithersburg, MD) dipped in an overnight culture (1 × 10^9^ CFU/mL) of HG003, *fumC::NΣ*, or heat-killed HG003 resuspended in PBS. PBS was used as a wounding control. Once infected with approximately 10^4^ CFU, flies were placed in pint cages and allowed one hour to recover. After one hour, those that had not woken up from the anesthesia were considered dead and discarded. Survival was monitored each day at approximately the same time for 15 days. 100 male and 100 female flies were used to determine mortality rates of *D. melanogaster*.

### 2.5. Drosophila melanogaster bacterial load determination

Separate pint cages were set up to determine bacterial load of individual flies at specific time points (0 d, 3 d, 10 d, 14 d). Forty virgin males and forty virgin females were infected as previously stated for each treatment group. Once infected, individuals were given one hour to recover from anesthesia. After one hour, individuals that had not woken up were considered dead and removed. This point was also considered the start of the time course, and flies were aged to the appropriate time. Following incubation, flies were washed in 1X PBS, and placed into 100 µL of 1X PBS and homogenized using an RNase-free disposable pellet pestle (Fisher Scientific, 12-141-368, Waltham, MA) attached to a Pellet Pestle™ cordless motor (Fisher Scientific, 12-141-361, Waltham, MA). Following homogenization, the cultures were serial diluted and plated onto TSA for enumeration.

### 2.6. Statistical analysis

Averages and standard deviations presented represent three biological replicates. Following *D. melanogaster* septic infection, a Kaplan-Meier survivorship analysis was performed to determine statistical significance. Significance was determined at p ≤ 0.05. Similarly, following bacterial enumeration from individual flies, statistical analysis was performed using a two-way ANOVA followed by a Tukey test. Significance was determined at p ≤ 0.05.

## 3. Results

### 3.1. Interruption of late TCA cycle genes increases tolerance to antibiotics

**Figure 1.**
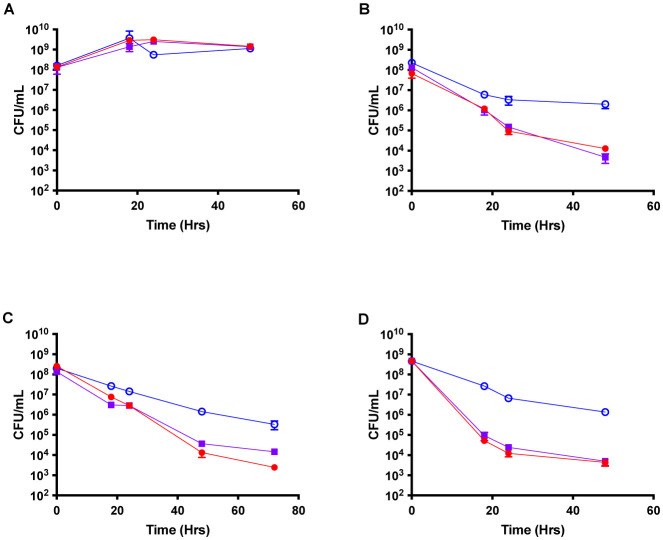
Interruption of TCA cycle genes leads to an increase in persisters when treated with different classes of antibiotics. **A)** The *fumC* mutant (blue) shows no difference in CFU in the absence of antibiotic compared to wild-type HG003 (red) and a *gltA* mutant (purple). When treated with 10 µg/mL ciprofloxacin (10x MIC; **B**), 10 µg/mL oxacillin (10x MIC; **C**), or 200 µg/mL daptomycin (50x MIC; **D**) the *fumC* mutant showed a 100-1,000-fold increase in persisters after 48–72 hours of antibiotic challenge.

To determine whether *S. aureus* persister cells can tolerate multiple classes of antibiotics, including those that may act in an energy-independent manner, antibiotic challenge experiments were performed with three antibiotics applied at 10- or 50-fold the minimal inhibitory concentration (MIC). As a control, survival in the absence of antibiotic treatment was monitored over 48 hours. All strains exhibit similar growth patterns, indicating there is no difference in survival when comparing TCA cycle mutants to wild-type *S. aureus* (Figure 1A). When ciprofloxacin, a DNA gyrase inhibitor, was added to the cultures at 10X MIC (MIC of 1 µg/mL), a decrease in CFU was observed in all strains (Figure 1B). However, *fumC::NΣ* exhibits approximately 1,000-fold more survival over a 48-hour timeframe than HG003 and *gltA::NΣ* (Figure 1B). In the presence of 10X MIC of oxacillin (MIC of 1 µg/mL), a b-lactam antibiotic binding to penicillin-binding proteins, live counts of all strains decreased by approximately 10-fold, with HG003 and *gltA::NΣ* continuing to decrease by three more orders of magnitude by 72 hours (Figure 1C). In contrast, *fumC::NΣ* displayed increased tolerance to oxacillin by 72 hours, as depicted by the characteristic plateau (Figure 1C). Finally, challenge with 50X MIC daptomycin (MIC of 4 µg/mL), an energy-independent pore-former, showed the most killing in the first 18 hours with HG003 and *gltA::NΣ* decreasing by approximately four orders of magnitude (Figure 1D). The *fumC::NΣ* strain again showed tolerance only decreasing by one order of magnitude by 48 hours (Figure 1D). When tested with multiple classes of antibiotics *fumC::NΣ* exhibited significantly more survival than wild-type HG003. Overall, these results demonstrate that interruption of the TCA cycle in the later steps results in increased tolerance to multiple classes of antibiotics.

### 3.2. Interruption of late TCA cycle genes increases tolerance to antimicrobial compounds

**Figure 2.**
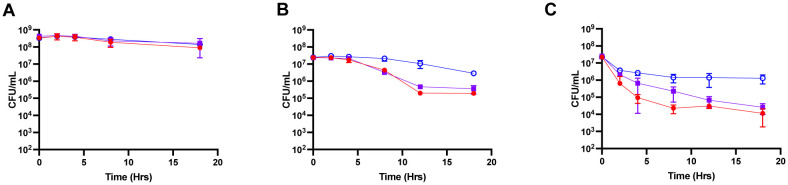
The *fumC* mutant has increased survival to antimicrobial peptides. Cultures were grown aerobically (1:10 volume to flask ratio in TSB) prior to dilution in PBS. Cultures were then left untreated (**A**) or challenged with 2.5 µg/mL LL-37 (**B**) or 5 µg/mL hβD3 (**C**). Surviving colonies were enumerated over the course of 18 hours. The *fumC::NΣ* strain (blue) exhibited increased survival after 18 hours when challenged with either antimicrobial peptide compared to HG003 (red) and *gltA::NΣ* (purple).

Due to the similarity in mechanisms of antibiotics and antimicrobial peptides, it was hypothesized that interruption of late TCA cycle genes would result in tolerance to antimicrobial peptides. Antimicrobial peptide challenge was performed with LL-37 and hβD3. Under no antimicrobial peptide challenge, all strains exhibit similar survival indicating that the phenotype is a result of AMP treatment (Figure 2A). When LL-37 was added to the cultures, there was a decrease in CFU for HG003 and *gltA::NΣ*, with HG003 showing 100-fold decrease by 18 hours (Figure 2B). Interestingly, by 12 hours *gltA::NΣ* had begun showing a less of a phenotype with less than 0.5 log difference in survival (Figure 2B). As expected, *fumC::NΣ* exhibited AMP tolerance with less than a fold-change by 18 hours (Figure 2B). Upon hβD3 challenge HG003 exhibited a 1,000-fold decrease by 18 hours (Figure 2C). *gltA::NΣ* exhibited less of a phenotype (less than 0.5 log difference in survival) and *fumC::NΣ* exhibited tolerance with only a fold-change by 18 hours (Figure 2C). Overall, these results indicate that interruption of the TCA cycle in the later steps results in increased tolerance to AMPs, indicating that TCA cycle mutants are able to form persisters not only to antibiotics but also AMPs.

### 3.3. The mechanism of action demonstrated by daptomycin and LL-37 is membrane potential dependent

It has been postulated that daptomycin is membrane potential dependent [22]. Similarly, LL-37 is also an ATP-independent pore former. CCCP has been used previously to dissipate membrane potential in Gram-negative bacteria. To investigate the mechanism of action of both daptomycin and LL-37, CCCP was utilized. To confirm that membrane potential was being disrupted in the same way in *S. aureus*, flow cytometry was utilized. Cultures of HG003 were either pre-treated with CCCP or left untreated and membrane potential was evaluated using the BacLight Bacterial Membrane Potential Kit. Cultures that were left untreated showed 92% of cells falling outside of the low membrane gate, indicating a higher membrane potential (Supplementary Figure 1). In contrast cultures that were pre-treated with CCCP showed 100% of cells falling within the low membrane gate, indicating a lower membrane potential (Supplementary Figure 1). This indicates that CCCP was dissipating membrane potential within *S. aureus*.

**Figure 3.**
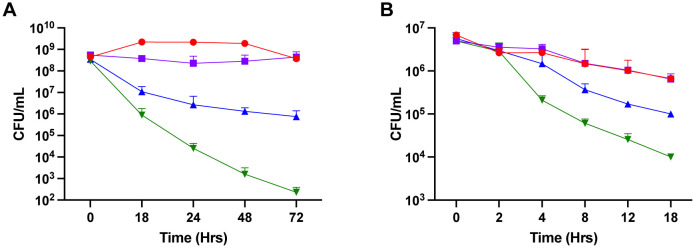
Disrupting membrane potential confers protection against daptomycin and LL-37. Carbonyl cyanide m-chlorophenylhydrazone (CCCP) was added at 20 µM for 10 minutes prior to daptomycin (200 µg/mL daptomycin, **A)** or LL-37 (2.5 µg/mL, **B)** challenge. Untreated HG003 (red) had similar survival after 18 hours compared to cultures treated with CCCP alone (purple). Challenging HG003 with either daptomycin (green) or LL-37 (green) results in five or three logs killing, respectively. Treating HG003 with CCCP prior to either daptomycin (blue) or LL-37 (blue) challenge increased survival by either three or one log(s), respectively.

Daptomycin is a membrane-acting antibiotic that has been hypothesized to be dependent on membrane potential [23]. To determine whether daptomycin and LL-37 killing is membrane-potential dependent, cultures were treated with CCCP to dissipate membrane potential. Following treatment with CCCP alone there was no significant change in survival after 72 hours, indicating that CCCP alone does not affect the viability of HG003 (Figure 3A). As seen previously, when HG003 was challenged with daptomycin, there was a decrease in CFU, resulting in a 10^6^-fold decrease by 72 hours (Figure 3A). However, when daptomycin challenge was combined with CCCP pretreatment, increased tolerance was exhibited compared to daptomycin challenge in the absence of CCCP, as shown by the plateau starting at 24 hours post-challenge (Figure 3A).

LL-37 has a similar mechanism to the proposed mechanism of daptomycin [15]. Therefore, cultures challenged with LL-37 were also treated with CCCP to dissipate membrane potential. As seen previously, CCCP alone has no significant effect on HG003 (Figure 3B). However, when LL-37 challenge was combined with CCCP treatment tolerance was exhibited, as shown by the increase in population survival (Figure 3B). These data suggest that both daptomycin and LL-37 are membrane potential dependent. Therefore, dissipating the membrane potential to eukaryotic levels increases persisters by approximately 1,000-fold and 10-fold, respectively.

### 3.4. Infection of D. melanogaster with fumC::NΣ leads to increased mortality

**Figure 4.**
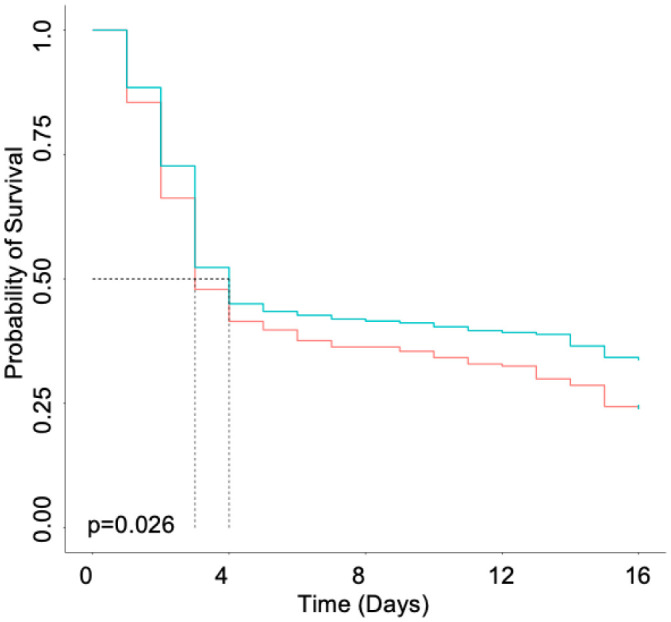
*D. melanogaster* males infected with *fumC::NΣ* exhibit increased mortality. Newly eclosed males (n = 100) were allowed to mature for 24 hours and then pricked in the thorax with a Tungsten needle dipped in an inoculum of wild-type HG003 (blue) or *fumC::NΣ* (red). Males infected with *fumC::NΣ* exhibit decreased survival when analyzed by a Kaplan-Meier survival curve, p = 0.026.

**Figure 5.**
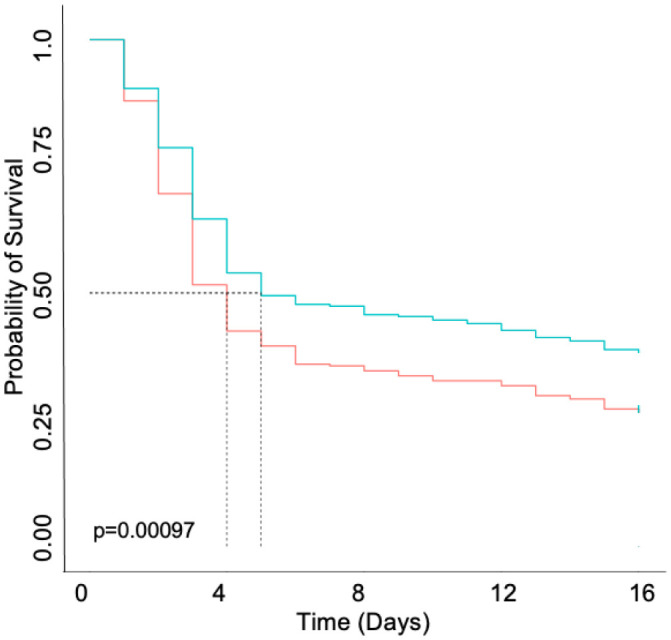
*D. melanogaster* females infected with *fumC::NΣ* exhibit increased mortality. Newly eclosed females (n = 100) were allowed to mature for 24 hours and then pricked in the thorax with a Tungsten needle dipped in an inoculum of wild-type HG003 (blue) or *fumC::NΣ* (red). Females infected with *fumC::NΣ* exhibit decreased survival when analyzed by a Kaplan-Meier survival curve, p = 0.00097.

*D. melanogaster* have been previously shown as a model for *S. aureus* infection through pricking the dorsal thorax. Previous studies have utilized MSSA and MRSA strains of *S. aureus* to investigate host-pathogen interactions [24],[25]. However, no long-term studies evaluating *S. aureus* persisters in *D. melanogaster* have been performed. Therefore, survival and bacterial enumeration were monitored in both males and females. Upon Kaplan-Meier survival analysis, it was determined that survival was statistically significant between males infected with HG003 and males infected with *fumC::NΣ*, resulting in males infected with *fumC::NΣ* dying sooner than those infected with wild-type HG003 (Figure 4; p = 0.026). Survival was also determined to be statistically significant between females infected with HG003 and females infected with *fumC::NΣ*, resulting in females infected with *fumC::NΣ* dying sooner than those infected with wild-type HG003 (Figure 5; p = 0.00097). However, there was no difference in survival between sexes challenged with the same strain (Figures S2 and S3; p = 0.12 and p = 0.49, respectively). Taken together these results suggest that TCA cycle mutants better survive the innate immune system within *D*. *melanogaster*.

### 3.5. D. melanogaster infected with fumC::NΣ exhibit an increased bacterial load

**Figure 6.**
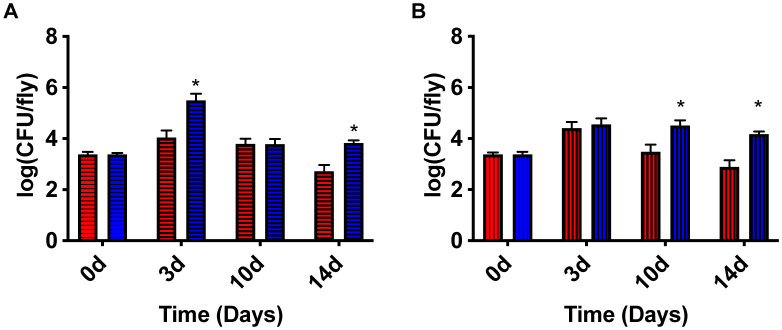
*D. melanogaster* infected with *fumC::NΣ* show an increased bacterial load. *D. melanogaster* were infected with either wild-type HG003 or *fumC::NΣ* using the pinprick method. Both males and females infected with *fumC::NΣ* show an increase in bacterial burden. Males infected with *fumC::NΣ* (blue horizontal line) have significantly higher bacterial burden at three days post infection and 14 days post infection when compared to males infected with HG003 (red horizontal line) (p =< 0.0001 and p = 0.0178; **A**). Females infected with *fumC::NΣ* (blue vertical line) have significantly more bacteria at 10 days post infection and 14 days post infection when compared to females infected with HG003 (red vertical line) (p = 0.0008 and p = 0.0212; **B**).

Due to the increased mortality exhibited by *D. melanogaster* infected with *fumC::NΣ* and the increased tolerance to AMPs exhibited by *fumC::NΣ*, it was postulated that these flies would have increased bacterial burden due to their inability to clear the infection. Persisters have been examined within a mouse biofilm infection; however, persister survival within a septic infection remain unstudied [26]. Males infected with *fumC::NΣ* showed an increased bacterial load at three days post infection (2 logs) and 14 days post infection (1 log; Figure 6A; p =< 0.0001 and p = 0.0178, respectively). Similarly, females infected with *fumC::NΣ* showed an increase in bacterial load at 10 days post infection (1 log) and 14 days post infection (2 logs; Figure 6B; p = 0.0008 and p = 0.0212, respectively). Previously, it was shown that increased sexual activity can reduce male immunity and seminal fluid components can reduce female immunity [21],[27]. Therefore, *Drosophila* that had not mated were used to examine whether there is a sex bias. However, in *Drosophila* infected with wild-type HG003 there was no significant difference found between sexes at any time point post infection (Figure S4A). In *Drosophila* infected with *fumC::NΣ* males exhibited higher bacterial load at three days post infection (p = 0.0034), but females exhibited higher bacterial load at 10 days post infection (Figure S4B; p = 0.0415). Overall, these results suggest that TCA cycle mutants increase mortality within *D. melanogaster* because of increased bacterial burden during the course of infection.

## 4. Discussion and conclusions

The current study supported the hypothesis that TCA cycle mutants exhibit increased persister formation to both antibiotics and components of the innate immune system. This can be seen when cultures were challenged with both antibiotics and AMPs (Figures 1 and 2). Without antibiotic challenge there was no difference in growth between strains; thus there was no growth defect associated with either *fumC::NΣ* or *gltA::NΣ*.

Persister formation within *S. aureus* is associated with ATP depletion [8]. Recently, it has been shown that *fumC::NΣ* exhibits depleted ATP in comparison with HG003 [13]. Therefore, this population should exhibit increased antibiotic tolerance. This phenotype can be seen by the increase in survival exhibited by the *fumC::NΣ* strain after both antibiotic and AMP treatment (Figures 2 and 4). Unsurprisingly, the *gltA::NΣ* strain did not exhibit the same phenotype. Previous work demonstrated that disruption of the *gltA* gene didn't have a reduction in ATP, likely from glutamate feeding into the TCA cycle at 2-oxoglutarate [13].

Generally, antibiotics and AMPs selectively act on prokaryotic cells because they require targets that only these organisms possess, such as 30S ribosomal subunits and DNA gyrase [28],[29]. Antibiotics that work in these manners rely on ATP to work by disrupting energy-dependent targets [30]. However, there are a few antibiotics and AMPs, such as daptomycin and LL-37, that have been proposed to be independent of ATP concentrations, and instead, dependent on membrane potential. Eukaryotic cells have a low membrane potential when compared with bacterial cells, which allows for ATP-independent antibiotics and AMPs to select for the higher potential bacterial cells [31]. Wild-type populations that were susceptible to daptomycin and LL-37 challenge alone exhibit tolerance when pre-treated with CCCP to dissipate membrane potential (Figure 3). This is indicative that persister formation may also be dependent on membrane potential. Previous work has shown that TCA cycle mutants have decreased membrane potential in comparison to wild-type populations [32]. Recent work has applied similar concepts with metabolism independent antibiotics when trying to eradicate persisters. In *E. coli*, persisters were successfully eradicated using combinational approaches with antibiotics that are strongly- and weakly-dependent on metabolism, thus eliminated non-persister and persister cells, respectively [33]. This work represents unique strategies that may have to be used to successfully eliminate persisters moving forward.

In addition to tolerance to antibiotics, *fumC::NΣ* exhibited increased tolerance to AMPs. Previously, LL-37 and hβD3 have been shown to be induced in cell culture by both MRSA and MSSA strains [34]. When treated with both LL-37 and hβD3, the *fumC::NΣ* mutant exhibited tolerance to both AMPs (Figure 2B and 2C). This is unlike a previous experiment in which similar concentrations of AMPs were added, but 100% of the bacteria were killed. This may be explained by a difference in starting CFU. Cultures within this study were treated at 10^7^ CFU/mL; however, cultures within the previous study were treated at 10^5^ CFU/mL. A difference of two logs in the starting CFU within the current study would decrease CFU/mL to below the level of detection. Persister formation is also growth phase dependent, with fewer persisters formed earlier in the growth phase. These differences offer a likely explanation for the differences in results.

In addition to evaluating persister formation in response to AMPs, *D. melanogaster* were also utilized to determine whether persisters provide a survival advantage *in vivo*. Flies were not infected with a defined amount of inoculum but starting CFU per fly was determined an hour after infection, typically flies were infected with 10^4^ CFU. Survival was monitored for 15 days, after which remaining flies were considered to have survived the infection. Preliminary data showed that flies remaining by 20 days had no signs of infection (data not shown), therefore, survival was determined at 15 days. It was determined that *D. melanogaster* infected with *fumC::NΣ* exhibited decreased longevity when compared with their counterparts infected with wild-type *S. aureus*. (Figures 4 and 5). Fruit flies share common innate immune components with humans, such as AMPs [24]. Therefore, it is possible that while this study tested the tolerance of persisters to human AMPs, this phenotype would continue across species. Interestingly, there was no sex bias observed between males and females infected with the same strain (Figures S2 and S3). It is widely known that mating influences not only female immune function but can also negatively impact male immune function in *D. melanogaster* [35]–[39]. However, the present study examined virgin males and females in order to eliminate the possibility of immune system suppression caused by mating. Therefore, when sexes infected with the same strain of *S. aureus* were compared, there was no difference in survival, indicating that immune suppression described previously is due solely to mating behavior.

It was also seen that bacterial load was higher in flies infected with *fumC::NΣ* than compared to those infected with wild-type *S. aureus* (Figure 6 A and B). The phenotype displayed indicates that *D. melanogaster* infected with *fumC::NΣ* have a hard time clearing the infection. This is illustrated by the decrease in CFU per fly associated with a wild-type *S. aureus* infection, whereas CFU per fly associated with a *fumC::NΣ* infection remains relatively constant during the course of infection. It is also interesting to note that when comparing males and females infected with *fumC::NΣ*, males exhibit increased bacterial load at three days post infection, but females exhibit increased bacterial load at 10 days post infection (Figure S4B). Males mature more rapidly in most insect species, including *Drosophila*. *D. melanogaster* males reach age of sexual maturity at two days post eclosion unlike females that take four days [40]. Mature males will also begin to court other males in the absence of females [41]. Therefore, it is not unlikely that the males used within this study exhibited immune suppression due to courting behavior between each other. This study utilized *D. melanogaster* due to production of AMPs similar to those found in humans [42]. While tolerance was observed to AMPs *in vitro* and survival was decreased *in vivo* in those flies infected with *fumC::NΣ*, it is also possible that the phenotype observed in the *D. melanogaster* is due to other components of the *D. melanogaster* immune system, such as recognition by peptidoglycan recognition proteins (PGRPs) ultimately leading to phagocytosis by plasmatocytes [43].

This study indicates that interruption of the TCA cycle, particularly in the later steps increases tolerance not only to antibiotics but also to AMPs. It also showed that persisters pose a problem for the innate immune system, specifically AMPs, and contribute to survival within a host. More research is needed to tease apart how persisters interact with other components of the innate immune system and contribute to increased mortality within a host.
