# Supplementary material for: Interruption of the tricarboxylic acid cycle in Staphylococcus aureus leads to increased tolerance to innate immunity
Source: AIMS Microbiol. 2021 Dec 15;7(4):513–27. doi: 10.3934/microbiol.2021031 (PMC8712538; doi:10.3934/microbiol.2021031)
Supplement: Supplementary file 1 [file microbiol-07-04-031-s001.pdf]

Microbiology

AIMS Microbiology, 7(4): 513–527.
DOI: 10.3934/microbiol.2021031
Received: 29 October 2021
Accepted: 13 December 2021
Published: 15 December 2021

http://www.aimspress.com/journal/microbiology

*Research article*

# Interruption of the tricarboxylic acid cycle in *Staphylococcus aureus* leads to increased tolerance to innate immunity

**Alexis M. Hobbs, Kennedy E. Kluthe, Kimberly A. Carlson, and Austin S. Nuxoll***

Department of Biology, University of Nebraska at Kearney, 2401 11th Ave, Kearney, NE 68849, USA

*  **Correspondence:** Email nuxollas@unk.edu; Tel: +13088658602; Fax: +13088658045.

## Supplemental

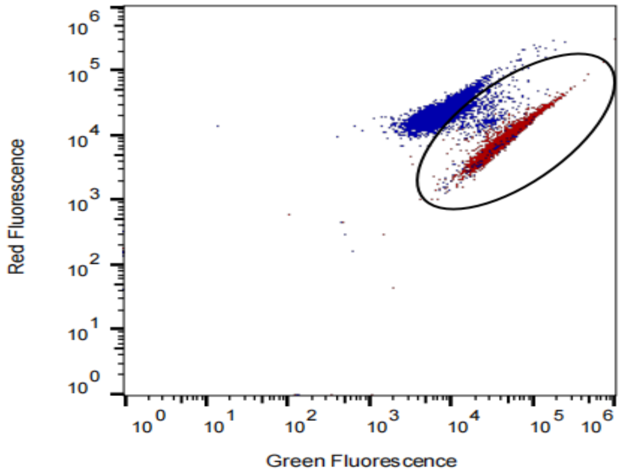


**Figure S1.** Treatment with CCCP dissipates membrane potential. HG003 was grown to mid-exponential phase and membrane potential was measured using BacLight Bacterial Membrane Potential Kit according to manufacturer's instructions. $DiOC_2(3)$ was excited at 488 nm, and emissions of the green and red fluorescence were detected with bandpass filters of 515/20- and 616/23-nm, respectively. A gate was drawn around low membrane potential cells with 100% of CCCP treated cells (red) and 8% of nontreated cells (blue) occurring within the gate.

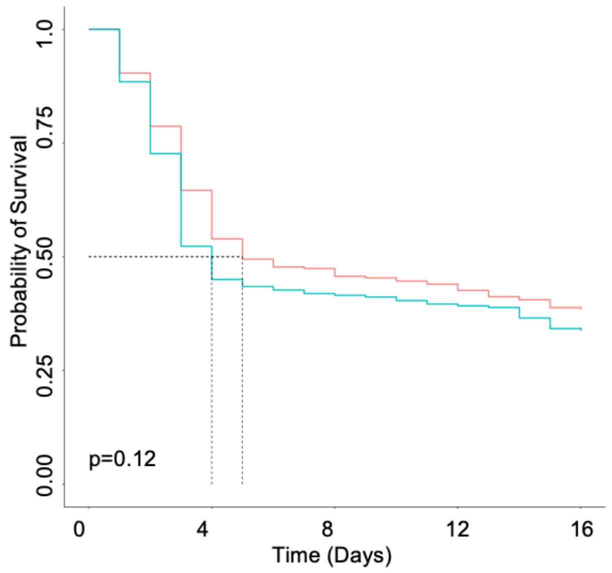


**Figure S2.** *D. melanogaster* infected with wild-type HG003 show no sex bias in their immune function. Newly eclosed *D. melanogaster* were separated and allowed to mature for 24 hours. Following maturation flies were pricked in the thorax with a Tungsten needle dipped in an inoculum of wild-type HG003. Upon Kaplan-Meier analysis there appears to be no difference in survival based on sex (males – blue, females – red), p = 0.12.

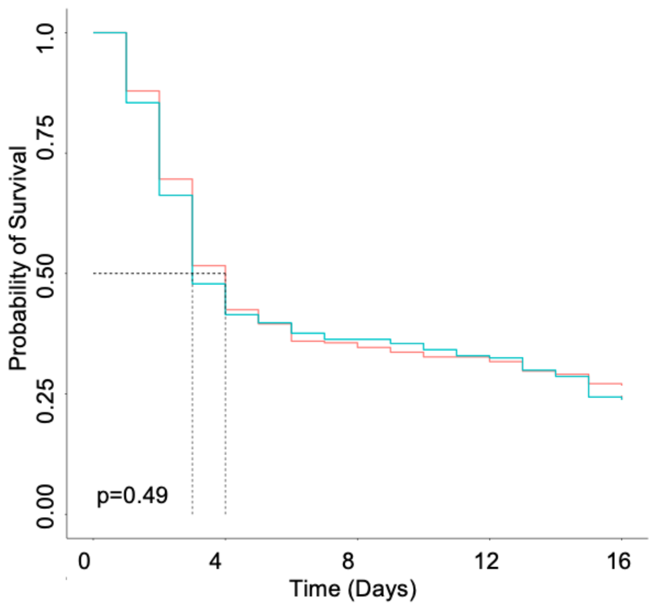


**Figure S3.** *D. melanogaster* infected with *fumC::NΣ* show no sex bias in their immune function. Newly eclosed *D. melanogaster* were separated and allowed to mature for 24 hours. Following maturation flies were pricked in the thorax with a Tungsten needle dipped in an inoculum of *fumC::NΣ*. Upon Kaplan-Meier analysis there appears to be no difference in survival based on sex (males – blue, females – red), p = 0.12.

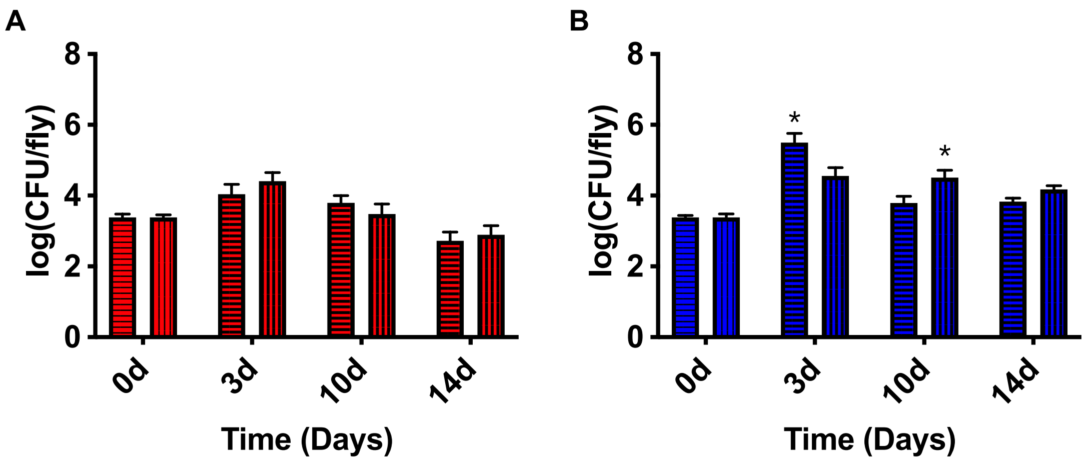


**Figure S4.** *D. melanogaster* infected with the same strain of *S. aureus* show no sex bias in bacterial load. *D. melanogaster* were infected with either wild-type HG003 or *fumC::NΣ* using the pinprick method. There is no difference in bacterial load based on sex when *D. melanogaster* are infected with wild-type HG003 (males-red horizontal line, females-red vertical line) (**A**). However, males infected with *fumC::NΣ* (blue, horizontal line) have higher bacterial load than females infected with *fumC::NΣ* (blue, vertical line) at three days post infection ($p = 0.0034$; **B**). In contrast, females infected with *fumC::NΣ* show a higher bacterial load than males infected with *fumC::NΣ* at 10 days post infection ($p = 0.0415$; **B**).

AIMS Press

© 2021 the Author(s), licensee AIMS Press. This is an open access article distributed under the terms of the Creative Commons Attribution License (http://creativecommons.org/licenses/by/4.0)
